# Profiling of normal and malignant breast tissue show CD44^high^/CD24^low^ phenotype as a predominant stem/progenitor marker when used in combination with Ep-CAM/CD49f markers

**DOI:** 10.1186/1471-2407-13-289

**Published:** 2013-06-14

**Authors:** Hazem Ghebeh, Ghida Majed Sleiman, Pulicat S Manogaran, Amer Al-Mazrou, Eman Barhoush, Falah H Al-Mohanna, Asma Tulbah, Khalid Al-Faqeeh, Chaker N Adra

**Affiliations:** 1 Stem Cell & Tissue Re-engineering Program, King Faisal Specialist Hospital & Research Centre, Riyadh, Saudi Arabia; 2 Stem Cell & Tissue Re-engineering Program, Departments of Comparative Medicine, King Faisal Specialist Hospital and Research Centre, Riyadh, Saudi Arabia; 3 Stem Cell & Tissue Re-engineering Program, Departments of Pathology, King Faisal Specialist Hospital and Research Centre, Riyadh, Saudi Arabia; 4 Stem Cell & Tissue Re-engineering Program, Department of Surgery, King Faisal Specialist Hospital and Research Centre, Riyadh, Saudi Arabia; 5 College of Medicine, Al-Faisal University, Riyadh, Saudi Arabia; 6 Transplantation Research Center (TRC), Brigham & Women's Hospital and Children’s Hospital Boston, Harvard Medical School, Boston,MA, USA; 7 Stem Cell & Tissue Re-engineering Program, Research Centre, King Faisal Specialist Hospital and Research Centre, PO Box 3354, Riyadh, (MBC 03), 11211, Kingdom of Saudi Arabia

**Keywords:** Normal breast, Stem cells, Breast cancer, Flow cytometry, CD10, ALDH, CD44^high^/CD24^low^, MUC-1, Mammary gland

## Abstract

**Background:**

Accumulating evidence supports cancer to initiate and develop from a small population of stem-like cells termed as cancer stem cells (CSC). The exact phenotype of CSC and their counterparts in normal mammary gland is not well characterized. In this study our aim was to evaluate the phenotype and function of stem/progenitor cells in normal mammary epithelial cell populations and their malignant counterparts.

**Methods:**

Freshly isolated cells from both normal and malignant human breasts were sorted using 13 widely used stem/progenitor cell markers individually or in combination by multi-parametric (up to 9 colors) cell sorting. The sorted populations were functionally evaluated by their ability to form colonies and mammospheres, *in vitro*.

**Results:**

We have compared, for the first time, the stem/progenitor markers of normal and malignant breasts side-by-side. Amongst all markers tested, we found CD44^high^/CD24^low^ cell surface marker combination to be the most efficient at selecting normal epithelial progenitors. Further fractionation of CD44^high^/CD24^low^ positive cells showed that this phenotype selects for luminal progenitors within Ep-CAM^high^/CD49f + cells, and enriches for basal progenitors within Ep-CAM^-/low^/CD49f + cells. On the other hand, primary breast cancer samples, which were mainly luminal Ep-CAM^high^, had CD44^high^/CD24^low^ cells among both CD49f^neg^ and CD49f + cancer cell fractions. However, functionally, CSC were predominantly CD49f + proposing the use of CD44^high^/CD24^low^ in combination with Ep-CAM/CD49f cell surface markers to further enrich for CSC.

**Conclusion:**

Our study clearly demonstrates that both normal and malignant breast cells with the CD44^high^/CD24^low^ phenotype have the highest stem/progenitor cell ability when used in combination with Ep-CAM/CD49f reference markers. We believe that this extensive characterization study will help in understanding breast cancer carcinogenesis, heterogeneity and drug resistance.

## Background

Breast cancer is the most common cancer in women and, despite various treatment regimens, many patients die from the disease. A subpopulation of tumor cells, called cancer stem cells (CSC), is believed to contribute to the failure of breast cancer therapy due to their reported resistance to chemotherapy [1,2] and radiotherapy [3]. Due to their self-renewal abilities, even a minute population of CSC can form tumor when isolated and injected into an appropriate mouse model, while the remaining cells fail to do so [4]. Breast CSC have been characterized as CD44^high^/CD24^low ^[5], or aldehyde dehydrogenase enzyme (ALDH)^high^[6]. However, how CSC relate to the different stem/progenitor cell populations of normal human mammary gland and whether CSC arise from normal mammary stem/progenitor or even differentiated cells, remains unanswered [4].

The normal mammary gland epithelium is composed of two types of epithelial cells: 1) basal contractile cells (mostly myoepithelial) that are in direct contact with the basement membrane and, 2) secretory luminal cells that face the lumen of ducts/lobules. The phenotype of the normal human mammary gland stem/progenitor cells has been described in various reports as being aldehyde dehydrogenase (ALDH)^high ^[6], CD10+ [7,8], CD44^high^CD24^low ^[5] or Ep-CAM+/MUC-1^neg ^[9]. Additionally, two other markers have become a standard combination for studying human normal mammary gland cells: EP-CAM (epithelial specific antigen, ESA) and CD49f (α-6-integrin) [10]. Previous reports indicate that Ep-CAM^high^ labels luminal epithelial cells where the Ep-CAM^high^/CD49f + fraction contains the luminal progenitors, while Ep-CAM^high^/CD49f^neg^ cells represent the differentiated luminal cells. On the other hand, Ep-CAM^-/low^/CD49f + phenotype characterize mainly the basal fraction of the human epithelial cells [8,11]. Whether the aforementioned stem/progenitor cell markers, described in different reports, identify overlapping cell populations and whether they are related to CSC remains unknown.

In this study, we demonstrated that human mammary epithelial cells with CD44^high^/CD24^low^ phenotype have the highest progenitor ability compared to all other stem/progenitor subpopulations. Furthermore, we have demonstrated that, in both normal and malignant breasts, there are multiple CD44^high^/CD24^low^ subpopulations. In the majority of breast cancer cases, CSC with CD44^high^/CD24^low^ phenotype existed in the Ep-CAM^high^/CD49f + fraction of cancer cells. Stem/progenitor markers should be used in combination with Ep-CAM/CD49f reference markers to indicate the pure/specific epithelial stem/progenitor cells. We believe this study may provide a better understanding of breast cancer carcinogenesis as well as facilitate the more accurate identification of CSC. Subsequently, these findings might help in monitoring and/or targeting of this population in the future.

## Methods and materials

### Patient selection and consenting

This study was conducted in accordance with the Helsinki Declaration and approved by the Research Advisory Council (RAC# 2080-045) of King Faisal Specialist Hospital and Research Centre (KFSH&RC). Normal human mammary gland tissues were obtained from 16 patients admitted to KFSH&RC who underwent reduction mammoplasty with no previous history of breast cancer. Breast cancer samples were obtained from 16 patients diagnosed with invasive ductal carcinoma of the breast (cases were ER + (n = 5), ER/Her2 (n = 3), Her2 (n = 3) and basal tumors (n = 5) ER = ER+/PR+/Her2^neg^, ER/Her2 = ER+/PR + or^neg^/Her2+, Her2 = ER^neg^/PR^neg^/Her2+, and basal = ER^neg^/PR^neg^/Her2^neg^). All patients signed an informed consent approved by KFSH&RC.

### Tissue processing and cell preparation

Processing of breast cancer tissues was performed after routine pathological examination. Gross tissue specimens were macro-dissected by pathologist and frozen sections were examined by hematoxylin staining to ensure that they contained carcinoma cells. Breast tissues (plastic surgery or breast cancer) were kept at 4°C in complete medium, (DMEM medium with 10% fetal bovine serum (FBS)) and processed within 1-2 hours. Tissues were minced, transferred to collagenase digestion medium (Stem Cell Technologies (SCT), Vancouver, Canada) and agitated with an Adams Nutator Mixer (Becton Dickinson, Franklin Lakes, NJ) at 37°C.

**Normal Tissue** pieces were digested overnight and cell suspensions were centrifuged at 800 g for 8 minutes at 4°C. Fat was aspirated and cell pellets were re-suspended in phosphate buffered saline (PBS). Cells were differentially centrifuged at 120 g for 2 minutes at 4°C to enrich for epithelial cells. The epithelial-enriched pellet was further digested with accutase (SCT) at 37°C (30-60 minutes) until organoids disaggregated into single cells. Epithelial cells were washed once with medium or PBS, filtered through a 70 μm mesh (BD Falcon, Bedford, MA, USA), re-suspended in freezing medium (composed of 90% FBS and 10% DMSO (sigma, St. Louis, Mo, USA)) and stored frozen under liquid nitrogen for later use.

**Tumor Tissues** were digested, as stated above, for 6 hours followed by centrifugation at 800 g for 8 minutes at 4°C. Cell pellets were further digested with accutase for 10-15 minutes and stored frozen in liquid nitrogen.

### Cell culture

Breast cancer cell lines were cultured in DMEM/F12 with the exception of SK-BR-3, which was cultured in McCoy's 5A medium. Both DMEM/F12 and McCoy's 5A media were supplemented with 10% fetal bovine serum and Antibiotics and Antimycotics (all from Invitrogen).

### Flow cytometry

Cells were thawed, washed and, if deemed necessary, 20 to 60 μl of DNase (SCT) was added to disaggregate clumps. Cells were depleted of CD31+ endothelial cells, whenever necessary, using MACS system (Miltenyi Biotec, Germany), as per manufacturer’s instructions. Cells were stained with panels of 8 different antibodies labeled with up to 8 different fluorescent dyes in addition to 4',6-diamidino-2-phenylindole (DAPI, Invitrogen) viability dye. The panels and antibody combination used are listed in Tables 1 and 2 respectively. An ALDH kit (Stem Cell Technologies) was used to stain ALDH population as per manufacture instructions. ALDH staining was performed first followed by the addition of the other antibodies as recommended by the ALDH kit.

**Table 1:** The panels of antibodies used to analyze the breast cells

	**Label**	**Panel 1**	**Panel 2**	**Panel 3**	**Panel 4**
		**(Ep-CAM**^**low**^**)**	**(Ep-CAM**^**high**^**)**	**(Other markers)**	**(Cancer)**
1	Pacific Blue	DAPI	DAPI	DAPI	DAPI
2	AmCyan	CD45	CD45	CD45	CD45
3	FITC	MUC-1*	ALDH	CD49f♣	CD31
4	PE	CD49f	CD49f	ABCB1	CD49f
5	Percp-Cy5.5			ABCG2ǁ	
6	PE-Alex 610	CD24	CD24	CD24	CD24
7	PE-Cy7	CD10	MUC-1	CXCR-4 ♦	CD10
8	APC	Ep-CAM	Ep-CAM	Ep-CAM	Ep-CAM
9	APC-Cy7	CD44	CD44	CD31	CD44

* Replaced with ALDH, CD31-FITC or others. ♦ Replaced in some experiments with c-kit (CD117) ♣Replaced with EPCR (CD201) in some experiments, || replaced with SSEA-4 in some experiments.

**Table 2:** Source, clone and dilution of Primary antibodies

	**Antibody**	**Company**	**clone**	**Added volume (per 1-6 x 10^6 cells) or dilution**
1	CD45-**AC**	BD	2D1	5 μL
2	MUC-1-**FITC**	BD	HMPV	5 μL
3	CD31-**FITC**	BD	WM59	5 μL
4	CD49f-**PE, FITC, PE-Cy5**	BD	GoH3	7 μL
5	EPCR (CD201) **PE**	BD	RCE-252	5 μL
6	SSEA-4-**Percp-Cy5.5**	Ebioscience	5E10	20 μL
7	ABCG2-**Percp-Cy5.5 or APC**	Biolegend	5D3	3 μL
8	CD24-**PE-Alex 610**	Invitrogen	SN3	5 μL
9	CD24 **FITC, or PE**	BD	ML5	5 μL
10	CD10-**PE-Cy7**	BD	HI10a	5 μL
11	CD117 (c-Kit)-**PE-Cy7**	Biolegend	104D2	5 μ
12	CXCR-4 (CD184) **PE-Cy7**	Biolegend	12G5	5 μ
13	Ep-CAM-**APC, FITC, or PE**	Miltenyi Biotec	HEA-125	7 μL
14	CD44-**APC-Cy7**	Biolegend	IM7	1 μL
15	CD31-**Alex 760**	Invitrogen	WM59	1 μL
16	Estrogen alpha-unlabelled	Abcam	polyclonal	1/300 dilution

* Indirectly labeled with secondary anti-rabbit pacific blue antibody.

Data were acquired using an LSR II Flow Cytometer while a FACSAria was utilized for cell sorting (both from BD, Biosciences, New Jersey, USA) using BD FACSDiva operating software according to guidelines set for analysis and sorting of stem cells by flow cytometry [12]. Positive staining was considered based on the negativity of an isotype control. A minimum of 10,000 events were recorded for all samples. Most phenotypic data were validated using the same antibodies with alternate labels.

### Gating strategy

We adopted a gating strategy to analyze single viable epithelial mammary cells. To this end, gates were established to exclude debris (using FSC and SSC) and hematopoietic cells (by gating on CD45^neg^ cells) and include only single viable cells (using the relation between FSC-A and FSC-W followed by gating on DAPI^neg^ cells). Gated cells were then examined with Ep-CAM and CD49f antibodies followed by a final gate to select stem/progenitor cells within each Ep-CAM/CD49f epithelial cell fraction. An example of the sequential gating is present in Additional file 1: Figure S1. ALDH^high^ positivity was selected based on staining parallel cells with DEAB inhibitor of ALDH activity (Additional file 2: Figure S2). Positivity for antibodies like CD44 and CD24 (as shown by the quadrant) was identified using isotype control (Additional file 2: Figure S2). CD44^high^ expression level was selected arbitrarily to include cells having fluorescence intensity (FI) units greater than 3000 FI (i.e. 2 minor ticks above 10^3^ FI). Similarly, CD24^low^ expression level was set to include cells having FI lower than 3000. Using these criteria we compared the abundance of CD44^high^/CD24^low^ cells in Ep-CAM^high^ or Ep-CAM^low^ populations. Due to lower expression of CD44 in luminal cells, compared with basal cells, we used the relative CD44^high^/CD24^low^ of Ep-CAM^high^/CD49f + cells during sorting and in functional assays (to obtain enough cells) and compared them with the bulk of Ep-CAM^high^/CD49f + cells (CD44^high^ expression levels were identified as cells with only 1 tick above 10^3^ FI i.e. 2000 FI). This increased the CD44^high^/CD24^low^ fraction of luminal Ep-CAM^high^/CD49f + cells from an average of 7% to 23%.

Primary breast cancer single viable cells were selected as above. In addition, lineage negative were selected using the CD10^neg^, CD31^neg^, and CD45^neg^ phenotypes and after excluding Ep-CAM^neg^/CD49f^neg^ mesenchymal cell fraction.

As a quality control, expression patterns were always cross referenced with known phenotypic data of breast cells subsets (e.g. basal and mesenchymal cells are CD24^neg^[13], mesenchymal cells are Ep-CAM^neg^/CD49f^neg^/MUC-1^neg^) This assured that our gating strategy was accurate.

### Immunohistochemistry

Routine immunohistochemistry of formalin-fixed, paraffin-embedded breast cancer samples were evaluated for Her2, estrogen receptor, and progesterone receptor status as reported previously [14].

Sorted stem/progenitor cell subpopulations were attached to glass slides and stained as described previously [15]. Briefly, cells were cytospin to glass slides by centrifugation at 800 rpm for 3 min. Slides were then air-dried overnight, acetone fixed for 15 min and stored at -80 C until stained. For staining, the cell membrane was permeabilized with 0.5% triton-X (Sigma) followed by overnight incubation with primary antibodies. After washing, Envision + polymer (ready to use; Dako) was used as a secondary antibody. Color was developed with 3,3′-diaminobenzidine (DAB) and instant hematoxylin (Shandon) was used for counterstaining.

Quantification of estrogen receptor (ER) positive cells, in normal sorted epithelial breast cells, was achieved by counting the number of ER + cells in several high magnification (x500) fields. The percentage was obtained by dividing the number of estrogen receptor positive cells by the total number of cells examined.

### Mammosphere formation assay

Cells were seeded in ultra-low attachment plates (Corning, Tewksbury, MA, USA) at a density of 1000 viable cells/well in 96-well plate in 120 μL/well of medium composed of DMEM/F12 medium supplemented with epidermal growth factor (20 ng/mL), hydrocortisone, (500 ng/mL), and Insulin (5 μg/mL), (all from Sigma) as well as B27 (1:50) and antibiotic/antimycotics (1:100) from Invitrogen (Grand Island, NY, USA) [9].

In addition to counting the number of mammospheres formed, we also measured the size of mammospheres by summing the volume (calculated as =4/3*π*r^3^ where r = radius of each mammosphere) of all formed mammospheres per cell group. This enabled us to assess the progenitor ability of the cells and to minimize the effect of mammosphere aggregation. Results are displayed as total sphere volume in microns^3^ or, where possible, mammosphere formation was normalized to the total mammosphere size of one of the Ep-CAM/CD49f main cell populations (i.e. Ep-CAM^low^/CD49f+, Ep-CAM^high^/CD49f + cells).

### Colony formation assay

Sorted cells were cultured at a density of 300 to 500 cells/cm^2^ on irradiated NIH 3T3 mouse cells seeded 1 day prior at a density of 50,000 cells/cm^2^. Cells were cultured in serum containing (2%) Ep-Wang medium [16] for 24 hours followed by medium exchange to serum free medium (Ep-sfm) [15]. Similar experiments utilizing Epi-Cult B medium (SCT) revealed comparable data. At the end of the experiment, colonies were counted under phase contrast microscope or directly after fixation with 4% PFA and staining with Giemsa (Fisher Scientific).

### Mouse xenotransplantation studies

All animal work, including anesthesia and euthanasia, was done in accordance to protocols approved by the Animal Care and Use Committee (ACUC) of KFSH&RC. Sorted (30, 000) MDA-MB-468 breast cancer cells were suspended in 50 uL FBS, mixed (1:1) with matrigel (BD Biosciences) and injected subcutaneously (MDA-MB-468).

### Statistical analysis

Significance in expression or mammosphere formation was determined by *T*-test using Excel software. P < 0.05 was used to indicate significance. Correlation coefficient was also calculated using Excel software. Error bars are presented as standard error of the mean (SEM).

## Results

In this study we used the Ep-CAM/CD49f antibody combination as a common reference to correlate CSC with normal stem/progenitors. We have characterized the normal breast Ep-CAM/CD49f epithelial fractions and their subpopulations then compared them with breast cancer cells.

### Breast epithelial stem/progenitor cells are limited to CD49f + cell fractions

**Figure 1:**
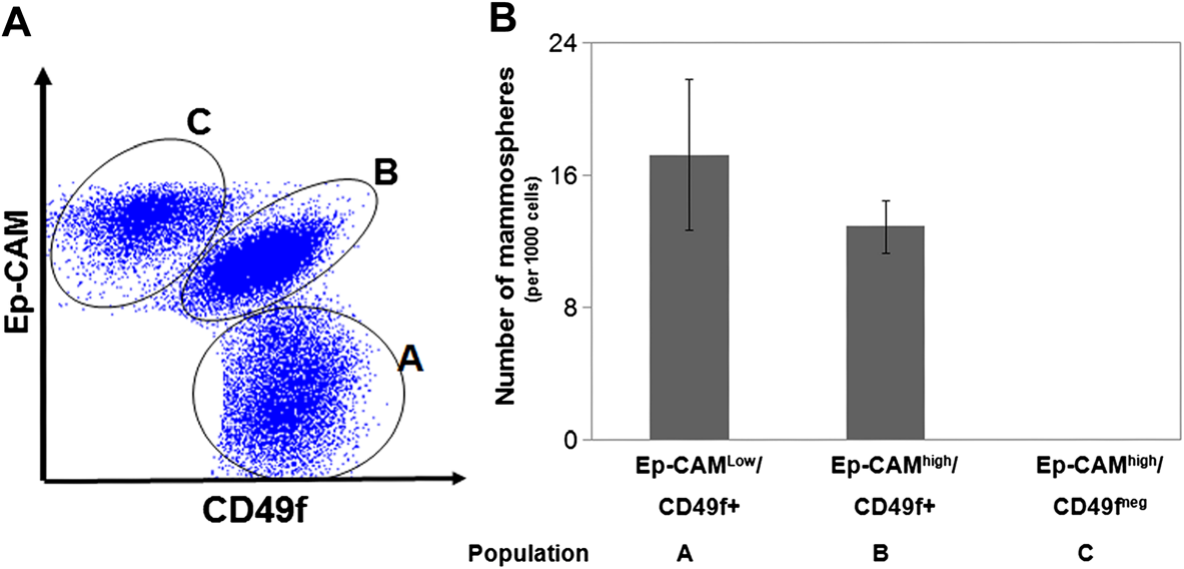
**Breast epithelial stem/progenitor cells are limited to CD49f + cell fractions. A**) A representative dot plot showing the three Ep-CAM/CD49f epithelial cell populations (designated as **A**, **B** and **C**) after exclusion of stromal (mesenchymal Ep-CAM^neg^/CD49^neg^, hematopoietic CD45+ and endothelial CD31+) cells and as analyzed by flow cytometry. **B**) Number of mammospheres formed from 1000 cells, of each of the three mammary epithelial cell populations, seeded in a 96-well low-attachment plate for 14 days (mean ± SEM, n = 2).

At First, we have re-established the stem/progenitor ability of the Ep-CAM/CD49f cell populations. After exclusion of stromal cells, Ep-CAM/CD49f staining displayed three distinct epithelial cell populations designated here for simplicity as A,B and C: Ep-CAM^-/low^/CD49f + (A), Ep-CAM^high^/CD49f + (B), and Ep-CAM^high^/CD49f^neg^ (C) (Figure 1A). Epithelial cell fractions were sorted and their stem/progenitor cell features were assessed using mammosphere and colony forming assays. Figure 1B shows that population A (Ep-CAM^-/low^/CD49f + cells) were the most efficient in forming mammospheres, consistent with being the source of mammary stem cells, followed by population B (Ep-CAM^high^/CD49f + cells). On the other hand, population C (Ep-CAM^high^/CD49f^neg^) cells did not form mammospheres. Consistently, in colony forming assays, population C did not form colonies while populations A and B gave typical basal (or mixed) and luminal colonies respectively (Additional file 3: Figure S3).

Altogether, these results confirm that basal Ep-CAM^-/low^/CD49f + (A) and luminal progenitor Ep-CAM^high^/CD49f + (B) cell fractions contain the stem/progenitor epithelial cells while Ep-CAM^high^/CD49f^neg^ (C) contain only differentiated cells. Therefore, we focused thereafter on CD49f + populations (A and B).

### CD44^high^/CD24^low^ epithelial cells showed the highest progenitor ability

Previous reports have used either one or two markers to identify stem/progenitor cells in the human mammary gland. However, to date, the relationship between different markers has not been well characterized. In order to link these stem/progenitor markers together we have simultaneously tested their expression levels in relation to Ep-CAM/CD49f profile using multi-parametric (up to 9 colors) cell sorting.

#### CD44^high^/CD24^low^ phenotype *within Ep-CAM*^*-/low*^*/CD49f + cells (A) enriches for basal progenitors*

**Figure 2:**
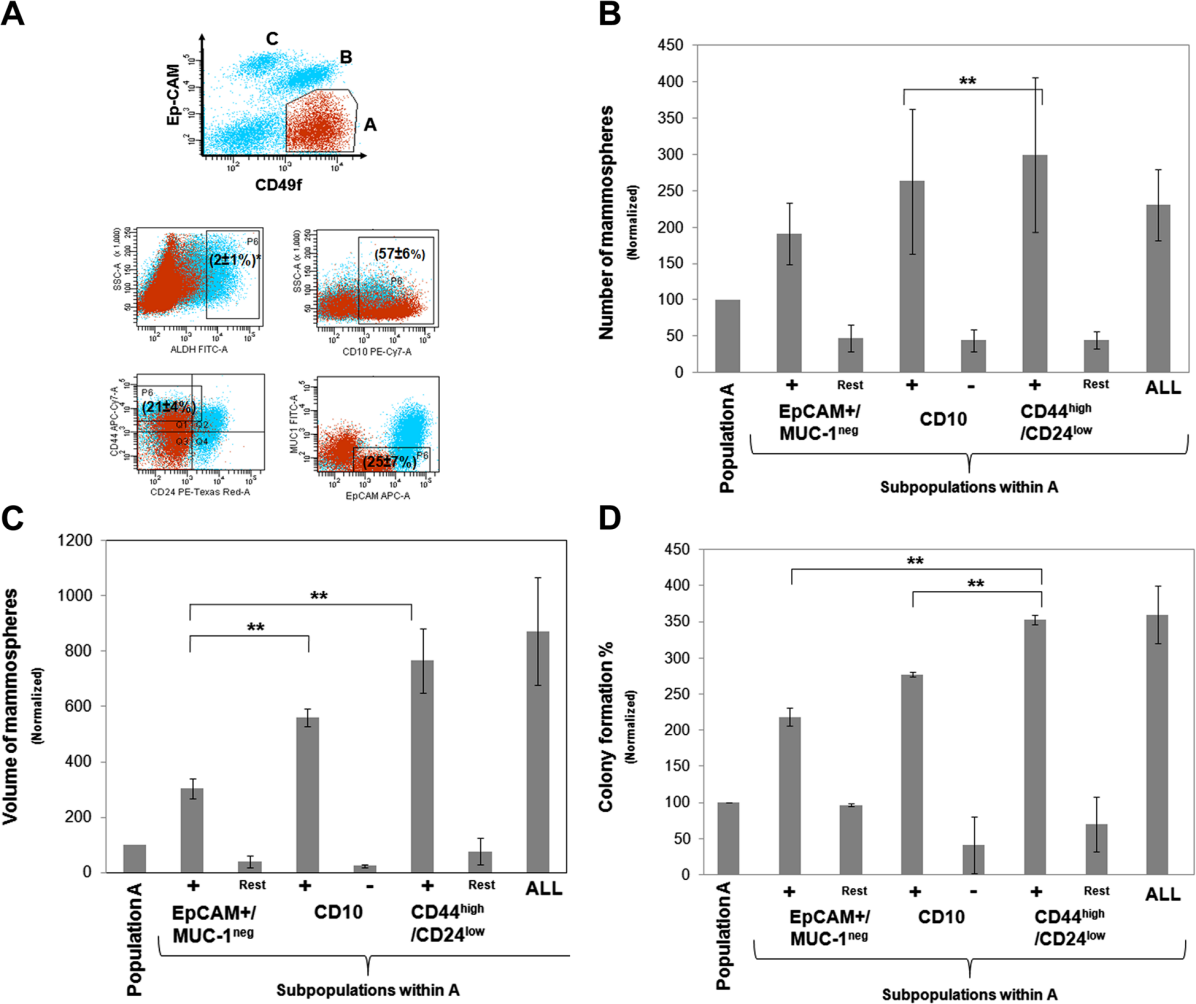
**CD44**^**high**^**/CD24**^**low **^**phenotype, within population A, enriches for basal progenitors. A**) A representative dot plot showing the expression of each stem/progenitor cell marker in the gated population A (Ep-CAM^-/low^/CD49f+, gated red dots) as analyzed by flow cytometry *numbers in brackets indicates average percentage (n = 10, mean ± SEM), quadrants show positivity while rectangles show "stem/progenitor" marker positive population. **B**&**C**) Number of mammospheres (**B**) and volume (**C**) of each stem/progenitor subpopulation within population A compared with the remaining bulk (rest) of population A (means ± S.E.M, n = 3). **D**) Colony formation assay for each cell subpopulation from population A (means ± S.E.M, n = 2). Data in **B**, **C** and **D** were normalized to unfractionated population A (Ep-CAM^-/low^/CD49f+), **indicate statistical significance (p < 0.05). ALL = the subpopulation within population A that express all the three stem/progenitor markers simultaneously.

Basal Ep-CAM^-/low^/CD49f + cells expressed: CD10 (57 ± 6%), CD44^high^/CD24^low^ (21 ± 4%) and Ep-CAM+/MUC-1^neg^ (25 ± 7%), but were ALDH^neg/or low^ (Figure 2A). In order to relate these markers to stem/progenitor cell function we sorted each stem/progenitor subpopulation within the Ep-CAM^-/low^/CD49f + group and examined their mammosphere and colony forming abilities (A representative gating strategy is presented in Additional file 1: Figure 1). All three stem/progenitor cell markers were effective in selecting for mammosphere forming cells. However, CD44^high^/CD24^low^ cells formed more mammospheres than other subpopulations, although the difference was not significant except between CD44^high^/CD24^low^ cells and CD10+ fractions (Figure 2B). Interestingly, when mammosphere size, which measure cell progenitor ability [9], was considered, CD44^high^/CD24^low^ formed significantly larger mammospheres than the other stem/progenitor subpopulations of Ep-CAM^-/low^/CD49f cells (Figure 2C). Consistently, colony forming assays showed cells with the CD44^high^/CD24^low^ phenotype have the highest number of basal (or mixed) colonies, while CD10^neg^ formed the lowest number of these colonies within the Ep-CAM^low^/CD49f + cell population (Figure 2D). Cells expressing all three stem/progenitor cell markers simultaneously (ALL) within Ep-CAM^-/low^/CD49f + cells did not form more mammospheres or colonies than CD44^high^/CD24^low^ cells suggesting that CD44^high^/CD24^low^ phenotype was accurate enough to select for basal progenitors (Figure 2C&2D).

These data indicate that within the basal Ep-CAM^-/low^/CD49f + (A) population, CD44^high^/CD24^low^ cells have the highest progenitor ability while, CD10^neg^ cells have the least progenitor ability (i.e. differentiated myoepithelial cells).

#### CD44^high^/CD24^low^ phenotype *within Ep-CAM*^*high*^*/CD49f + cells (B) selects for luminal progenitors*

**Figure 3:**
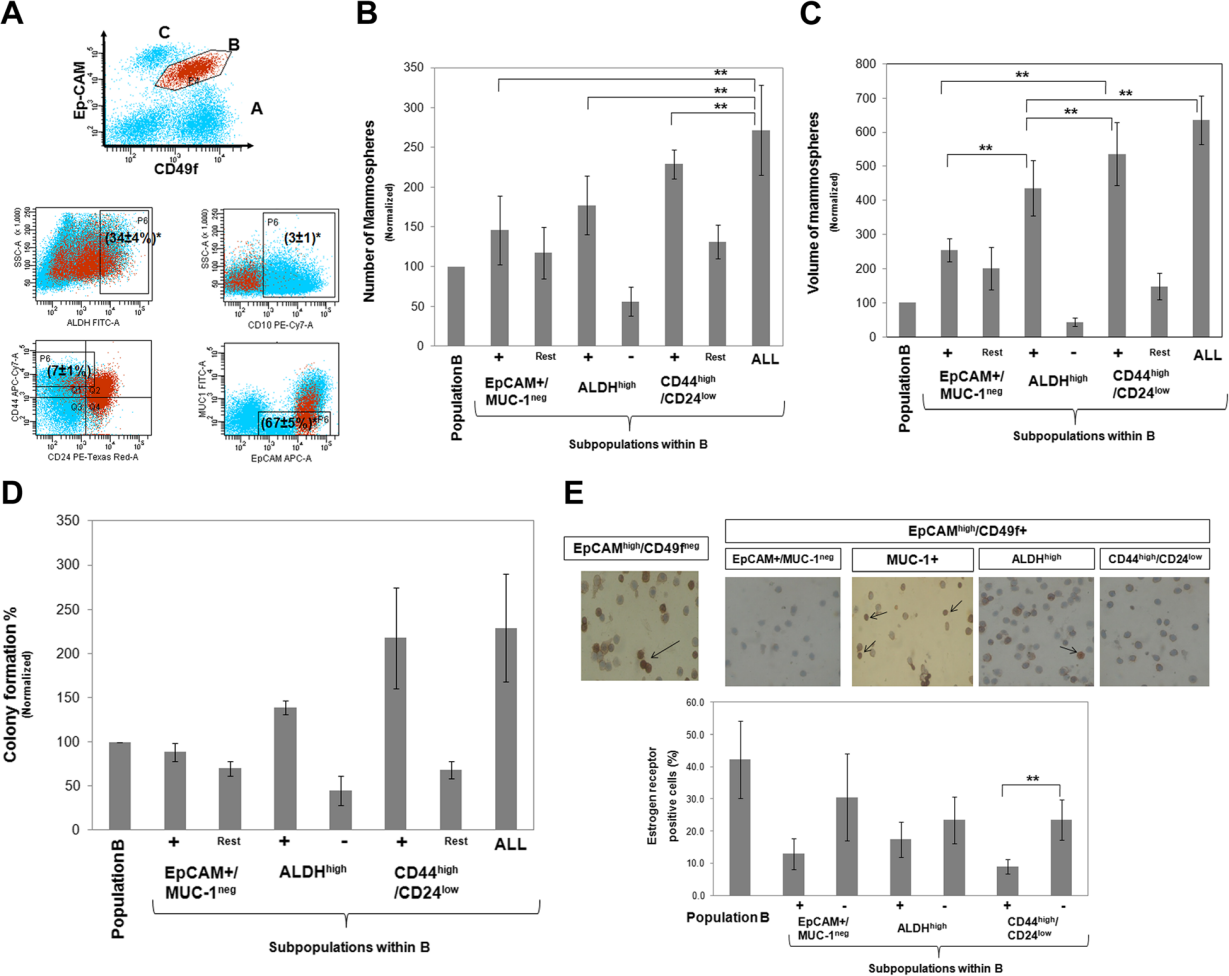
**CD44**^**high**^**/CD24**^**low **^**phenotype, within population B, selects luminal progenitors. A**) A representative dot plot showing the expression of each stem/progenitor cell marker in population B (Ep-CAM^high^/CD49f + population,gated red dots) as analyzed by flow cytometry, *numbers in brackets indicates average percentage (n = 10, mean ± SEM). Quadrants show positivity while rectangles show "stem/progenitor" marker positive population. **B**&**C**) Mammosphere formation measured by either number (b) or volume (**C**) of each stem/progenitor subpopulation, compared with the remaining bulk (rest) of population B (means ± S.E.M, n = 3). **D**) Number of colonies formed of each cell subpopulation from population B (means ± S.E.M, n = 2). Data in **B**, **C** &**D** are normalized to unfractionated population B, **indicate statistical significance (p < 0.05). **E**) Top) Representative image (x400,) of immunohistochemistry for estrogen receptor (nuclear, brown) in each cell subpopulation of population B compared with population C (Ep-CAM^high^/CD49f^neg^) cells as a positive control. Gills hematoxylin (nuclear, blue) was used as a counterstain. Bottom) Quantification of estrogen receptor positive cells in each subpopulation of population B. ALL = the subpopulation within population B that express all the three stem/progenitor markers simultaneously.

Luminal Ep-CAM^high^/CD49f + (B) cells expressed: ALDH^High^ (34 ± 4%), CD44^high^/CD24^low^ (7 ± 1%) and Ep-CAM^high^/MUC-1^neg^ (67 ± 5%) markers, while cells in this population were mostly CD10^neg^ (Figure 3A). These stem/progenitor marker(s) positive subsets, within the main Ep-CAM^high^/CD49f + population, were sorted to evaluate their mammosphere and colony forming abilities. Results show that cells with ALL followed by cells with CD44^high^/CD24^low^ phenotype demonstrated the highest mammosphere forming ability when both the number (Figure 3B) and the size of mammospheres were considered (Figure 3C), while ALDH^neg/low^ showed the least ability to form mammospheres or colonies (Figure 3B&C). Likewise, ALL and CD44^high^/CD24^low^ cells gave the highest percentages of colony forming cells (almost all luminal), although the difference was not statistically significant from other subpopulations (Figure 3C).

This demonstrates that luminal cells having the CD44^high^/CD24^low^ phenotype have the highest progenitor ability when used in combination with CD49f expression status.

#### CD44^high^/CD24^low^ cells *within Ep-CAM*^*high*^*/CD49f + population correlates with ER negative status*

Luminal mammary epithelial cells have a population of estrogen receptor (ER) positive cells that become the majority in hormone receptor positive breast cancers. On the other hand, in the normal breast, proliferating luminal cells are estrogen receptor negative [17]. Therefore, in order to identify these ER negative cells (presumably proliferating progenitor cells) we investigated the ER status of cells expressing stem/progenitor cell markers within the luminal Ep-CAM^high^/CD49f + (B) population. Immunohistochemistry of sorted cells revealed that within population B, cells with CD44^high^/CD24^low^ phenotype had the lowest number of ER positive cells, consistent with their progenitor ability (Figure 3E). Cells from Ep-CAM^high^/CD49f^neg^ population (population C), known to have the highest percentage of ER positive cells, were used as a positive control while cells from the Ep-CAM^-/low^/CD49f + population were used as a negative control [11]. These data show correlation between CD44^high^/CD24^low^ and estrogen receptor negativity in normal mammary epithelial cells within Ep-CAM^high^/CD49f + cells (B), which is consistent with their progenitor ability.

#### CD44^high^/CD24^low^ epithelial cells *within Ep-CAM*^*lhigh*^*/CD49f*^*neg*^*cells (population C) lack stem/progenitor ability*

Finally, we measured the expression level of the breast stem/progenitor cell markers in the Ep-CAM^high^/CD49f^neg^ cell population (C). Both CD44^high^/CD24^low^ and Ep-CAM+/MUC-1^neg^ phenotypes were expressed at 11 ± 3% and 16 ± 3% respectively (Additional file 4: Figure S4). On the other hand, there were neither ALDH^high^ (majority of cells were ALDH^low^) nor CD10+ cells in this population. Even though Ep-CAM^high^/CD49f^neg^ cells expressed CD44^high^/CD24^low^ and Ep-CAM+/MUC-1^neg^ markers, they did not form mammospheres or colonies *in vitro*. This emphasizes that CD44^high^/CD24^low^ and Ep-CAM+/MUC-1^neg^, previously described stem/progenitor markers, could not select for progenitor cells when used alone, and that they should to be used in combination with Ep-CAM/CD49f profile.

These results altogether, demonstrate that there are multiple subpopulations of progenitor cells within each Ep-CAM/CD49f cell group. The CD44^high^/CD24^low^ cells in both basal and luminal CD49f + fractions had the highest progenitor ability in each cell type respectively. Ep-CAM^high^/CD49f^neg^, although expressing some of the stem/progenitor cell markers, were differentiated cells as they failed to form colonies and mammospheres *in vitro* (data summarized in Table 3).

**Table 3:** Characterization of Ep-CAM/CD49f populations and the stem/progenitor cell markers they express

**Population**	**Lineage**	**Mammosphere formation**	**Has colony forming cells**	**Expressed "stem cell" markers**			
				**ALDH**	**CD10**	**CD44**^**high**^**/CD24**^**low**^	**Ep-CAM+/MUC-1**^**neg**^
Ep-CAM^neg^/CD49f^neg^	Mesenchymal	√	√	+++	++	+++	**-**
Ep-CAM^high^/CD49f^neg^	Luminal	NO	NO	**-**	**-**	+	+
Ep-CAM^high^/CD49f+	Luminal	√	√	+++	**-**	+	+++
Ep-CAM^low^/CD49f+	Myoepithelial	√	√	**-**	+++	++	++

#### Other “stem/progenitor” cell markers do not show any stem/progenitor enrichment ability over CD49f + cells

Besides the above examined common breast stem/progenitor cell markers, we evaluated additional markers that have been associated with stem/progenitor cells in the breast (CD133, CXCR-4, SSEA-4, c-kit, EPCR, ABCB1 and ABCG2), within the Ep-CAM and CD49f cell fractions. All the examined stem/progenitor cell markers were expressed by Ep-CAM^high^/CD49f + luminal progenitor cells, or Ep-CAM^-/low^/CD49f + basal cells. Functionally, none of these markers could further enrich for stem/progenitor cells over the main Ep-CAM^low^/CD49f + basal or Ep-CAM^high^/CD49f + luminal populations, as assessed by mammosphere formation (Additional file 5: Figure S5) and colony forming assays (data not shown). This indicates that these putative stem/progenitor cell markers did not enrich for stem/progenitor cells above CD49f + alone.

#### Cancer stem cells can best be enriched using combination of CD44^high^/CD24^low^ and Ep-CAM^high^/CD49+ markers

Subsequently, we sought to compare the stem/progenitor cell populations between normal mammary epithelial cells and breast cancer cells.

#### The majority of breast cancer cells have luminal phenotype

**Figure 4:**
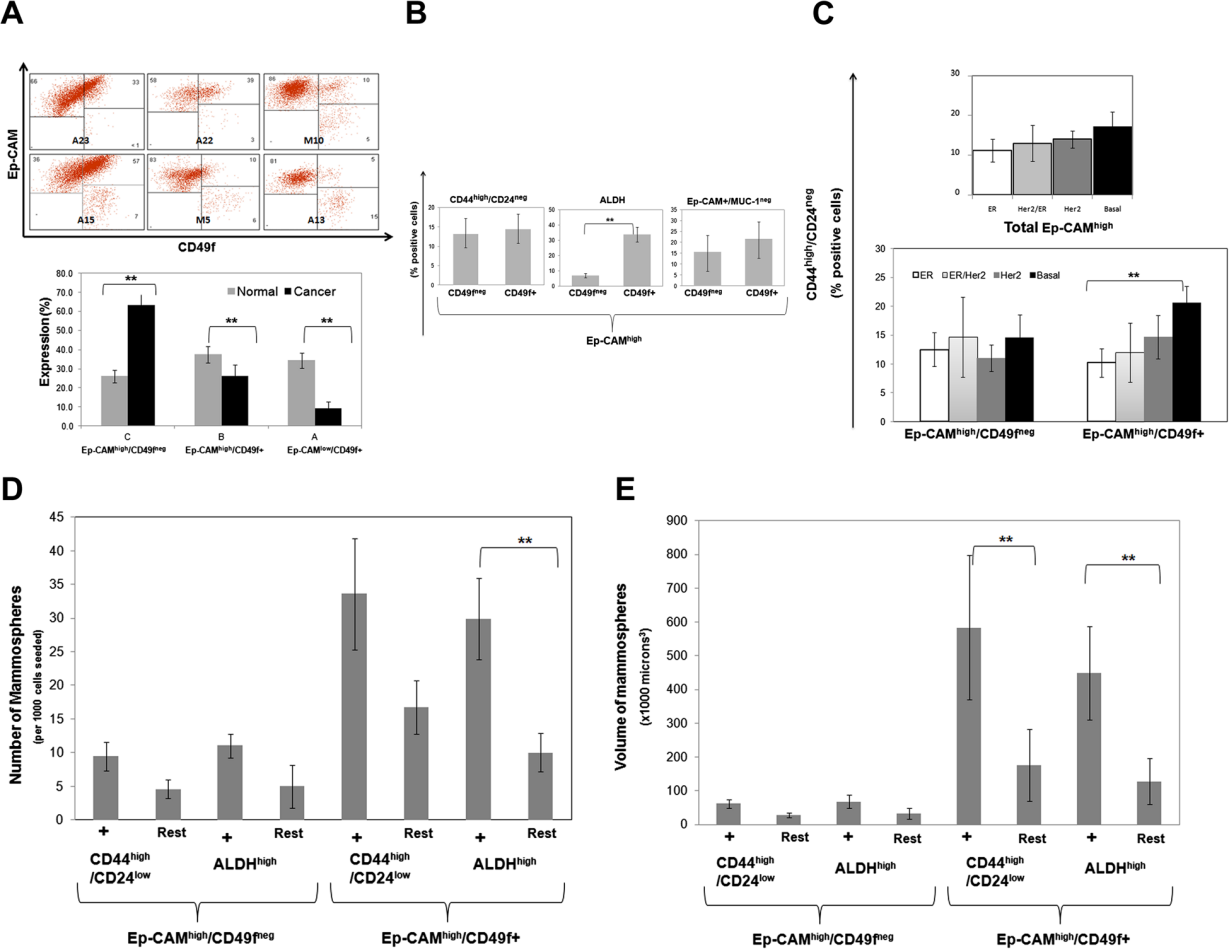
**CSC are abundant in primary CD44**^**high**^**/CD24**^**low**^**/Ep-CAM**^**high**^**/CD49+ cancer cells. A**) Representative dot plots for Ep-CAM/CD49f profile of tumor cells from different breast cancer patients as analyzed by flow cytometry (top) and histogram showing percentage of each Ep-CAM/CD49f population (n = 9 for normal breast and n = 13 for breast cancer samples). Lineage negative (CD45^neg^, CD10^neg^, CD31^neg^) cancer cells were gated followed by exclusion (gating out) of Ep-CAM^neg^/CD49f^neg^ mesenchymal fraction. Numbers in the corners indicate percentage of cells in each quadrant. **B**) Expression level of each of the studied stem/progenitor cell markers in Ep-CAM^high^/CD49f^neg^ and Ep-CAM^high^/CD49f + cell fractions in 16 breast cancer patients gated as in A. **C**) Percentage of CD44^high^/CD24^low^/Ep-CAM^high^ cancer cells (top) and after fractionation into CD49f^neg^ or CD49f + cells (bottom). Cases were stratified to four subtypes of breast cancers based on their estrogen receptor (ER), progesterone receptor (PR) status, as well as overexpression of Her2 as following: ER = ER+/PR+/Her2^neg^, ER/Her2 = ER+/PR+/Her2+, Her2 = ER^neg^/PR^neg^/Her2+, and basal = ER^neg^/PR^neg^/Her2^neg^, **D**&**E**) Mammosphere formation, measured by either number (**D**) or size (**E**) of tumor cells sorted for the stem/progenitor markers CD44^high^/CD24^low^ or ALDH^high^ and expressed within Ep-CAM^high^/CD49f^neg^ or Ep-CAM^high^/CD49f + breast cancer cell subpopulations (means ± S.E.M, n = 7). **indicates statistical significance.

While the standard profile for normal human mammary epithelial cells depends on the expression of Ep-CAM and CD49f, such data is currently not available for breast cancer cells. Therefore, we compared the Ep-CAM/CD49f expression patterns of normal mammary epithelial cells with primary tumor cells obtained from breast cancer patients. Our results show a clear drift in primary breast cancer cells towards population C (Ep-CAM^high^/CD49f^neg^), which almost doubled, while population A (Ep-CAM^-/low^/CD49f+) decreased dramatically in cancer cells - to less than one fourth of its normal counterpart (Figure 4A). As population A was barely present among the breast cancer cells, and existed in few patient samples, we focused on population B and C (i.e. Ep-CAM^high^/CD49f + and Ep-CAM^high^/CD49f^neg^ respectively) as they constituted the vast majority, if not all, of the tumor cells in primary breast cancer samples.

#### Both CD49f^neg^ and CD49f + cancer cells express stem/progenitor markers

We examined more closely the expression of the stem/progenitor cell markers in population C (Ep-CAM^high^/CD49f^neg^) and population B (Ep-CAM^high^/CD49f+) of tumor cells. We found no significant difference in the percentage of CD44^high^/CD24^low^ tumor cells among population C (Ep-CAM^high^/CD49f^neg^) and population B (Ep-CAM^high^/CD49f+) (Figure 4B). In contrast, the vast majority of ALDH^high^ cells were among the CD49f + stained cells. This clearly shows a phenotypic similarity in the distribution of stem/progenitor cell markers between primary breast cancer cells and normal epithelial cells. In contrast, unlike normal epithelial cells, there was no statistically significant difference in the percentage of Ep-CAM+/MUC-1^neg^ cells between Ep-CAM^high^/CD49f^neg^ and Ep-CAM^high^/CD49f + cells, implying an alteration in the MUC-1 expression upon carcinogenesis (Figure 4B). We further stratified the 16 breast cancer samples analyzed into the four main subtypes of breast cancer: ER (luminal A), ER/Her2 (luminal B), Her2 and Basal. There was no statistically significant difference in the expression of the stem/progenitor markers between the four types of breast cancer, probably due the small number of samples analyzed (data not shown). However, despite a small sample size, there was significant difference (P = 0.01) in CD44^high^/CD24^low^ expression among Ep-CAM^high^ breast cancer cells between ER and Basal subtypes of breast cancer. This difference was only present among CD49f + cancer cells (Figure 4C). This suggests that CD49f, if used in combination, with CD44^high^/CD24^low^ might be able to link stem/progenitor cell markers with breast cancer subtypes.

#### Cancer stem cells are abundant in primary CD44^high^/CD24^low^/Ep-CAM^high^/CD49+ cancer cells

We then functionally tested the stem/progenitor cell ability of the above examined subpopulations. We assessed the mammosphere formation of sorted CD44^high^/CD24^low^ or ALDH^high^ cells that were further fractionated from either population C (Ep-CAM^high^/CD49f^neg^) or population B (Ep-CAM^high^/CD49f+) cancer cells. Stem/progenitor cell subpopulations within CD49f + fractions of cancer cells formed more mammospheres than their CD49f^neg^ cell counterparts (Figure 4D). In addition the size of the mammospheres formed in CD49f + cancer cells were considerably larger than CD49f^neg^ cancer cells (Figure 4E). The CD44^high^/CD24^low^ cancer cells tended to form more mammospheres than ALDH^high^-although the difference was not statistically significant. This demonstrates that CSC can best be enriched by selecting for tumor cells with the CD44^high^/CD24^low^ or ALDH^high^ phenotypes within Ep-CAM^high^/CD49f + cancer cells.

#### Breast cancer cell lines are mostly Ep-CAM^high^/CD49+

Breast cancer cell lines are frequently used as a model to study breast cancer cells. Therefore, we have examined the phenotype of 9 commonly used breast cancer cell lines using the Ep-CAM/CD49f reference markers. Interestingly, similar to primary breast cancer cells 6 out of 9 cell lines had the Ep-CAM^high^ luminal phenotype (Additional file 6: Figure S6A). We then examined the expression levels of the stem/progenitor cell markers in these cell lines. Importantly, there was a correlation between the percentage of cells with CD44^high^/CD24^low^ phenotype and ER negativity status (correlation coefficient 0.63) (Additional file 6: Figure S6B).

Among all tested cell lines: MDA-MB-468 remarkably expressed the four major stem/progenitor cell markers previously examined i.e. CD44^high^/CD24^low^, ALDH^high^, Ep-CAM+/MUC-1^neg^ and CD10 (Additional file 6: Figure S6B). We therefore decided to use this cell line as a model to compare the four stem/progenitor markers. We sorted then injected low cell numbers from each stem/progenitor subpopulation of MDA-MB-468 cells into NOD/SCID mice. After 4 weeks only CD44^high^/CD24^low^ and ALDH^high^ cells formed tumors (in 4/4 of the injected mice). This shows that CD44^high^/CD24^low^ were comparable to ALDH^high^ in enriching for cancer stem cells in breast cancer cell lines.

These results collectively show that CD44^high^/CD24^low^ were comparable to ALDH^high^ phenotype in selecting for cancer stem cells both in primary, as well as established breast cancer cell lines. However, while these stem cell markers can be used alone in cell lines (vast majority are CD49f+), the primary CD44^high^/CD24^low^ breast cancer cells existed in both CD49f^neg^ and CD49f + cancer cell fractions. Therefore, in primary breast cancer samples, stem cell markers should be used in combination with Ep-CAM/CD49f antibodies.

## Discussion

The presence of stem/progenitor cell populations in the human breast has been well documented in several reports [8,9,18,19]. Several markers have been used to identify this population including Ep-CAM^-/low^/CD49f + [8], ALDH + [6], CD44^high^/CD24^low^[20], CD10+ [7], or Ep-CAM+/MUC-1^neg^[9]. However, to date there is no study with detailed comparison between these markers. Our approach was to compare phenotypically and functionally most of the previously reported stem/progenitor cell markers side-by-side in reference to Ep-CAM/CD49f profile. We found for the first time that CD44^high^/CD24^low^ mammary cells exhibited the highest stem/progenitor ability, both in normal and malignant breast cells, when combined with Ep-CAM/CD49f markers. We have used multi-parametric (up to 9 colors) fluorescence-activated cell sorting (FACS) coupled with several *in vitro* and *in vivo* assays to compare the progenitor/tumorigenic ability of the different stem/progenitor subpopulations of the human breast. Importantly, in this study, we have used uncultured/unmanipulated cells in contrast to several previous reports based on cells cultured for 3 days prior to analysis [8,10].

Since the pioneering work of Al-Hajj et al [5] on the phenotype of CSC as CD44^high^/CD24^low^, multiple studies have suggested that these markers did not correlated with the survival of breast cancer patients [21-23]. We have demonstrated in this study that CD44^high^/CD24^low^ from CD49f + cancer cells formed more mammospheres than CD49f^neg^ cancer cells. This indicates that it is necessary to use CD49f in combination with CD44^high^/CD24^low^ panel. In agreement, Cariati et al [24] have shown that only CD49f + MCF-7 form mammospheres and induce tumors in mice, and not CD49f^neg^ cells. Interestingly, very recent clinical data (utilizing large sample size of breast cancer patients) by Ali et al [25] have shown that CD44^high^/CD24^low^ or ALDH^high^ in combination with the CD49f positivity correlate with patient survival.

We have reported an abundance of CD44^high^/CD24^low^ in the human mammary gland of 21 ± 4% in Ep-CAM^low^/CD49f + and 7 ± 1% in Ep-CAM^high^/CD49f + cell populations. A recent study has reported that CD44^high^/CD24^low^ subpopulation is restricted to Ep-CAM^low^/CD49f + fraction of cells [26]. This discrepancy is most likely due to the CD44^high^ gating used. To our knowledge, there is no standard criterion in the literature to describe CD44^high^ and CD24^low^ gates (The criteria we used is clearly described in the methods and materials section). However, regardless of the gate used, we have established here that the relative fraction CD44^high^/CD24^low^ among Ep-CAM^high^/CD49f + cell population was able to enrich for colony forming cells more than 2 times the bulk of Ep-CAM^high^/CD49f + cells. This indicates that the gate for CD44^high^/CD24^low^ does not have to be the same for Ep-CAM^high^ and Ep-CAM^low^ in order to select for epithelial progenitors. This further supports the importance of using CD44^high^/CD24^low^ phenotype in combination with Ep-CAM/CD49 reference markers.

In this report, we have demonstrated that the majority of cancer cells showed a luminal Ep-CAM^high^ phenotype, with very small percentage of cancer cells of Ep-CAM^-/low^/CD49f + phenotype. Similarly, 6 out of 9 breast cancer cell lines had Ep-CAM^high^ phenotype. These findings are consistent with the previously described luminal phenotype of cancer cells based on strong correlative evidence showing breast cancer cells express luminal markers (such as MUC-1, Keratins 18 and 19 [27]), and lack basal markers (like CD10 and α-SMA [28]). In addition, Ince et al [29] established that transformed luminal cells (BPLER) were able to form tumors from as little as 10^2^ cells, while as many as 10^6^ cells were needed to form tumor from (HMLER) basal enriched transformed cells. This indicates that luminal cells are more tumorigenic than basal cells. In addition, Fillmore et al [13] have shown that CD44^high^/CD24^low^ within Ep-CAM + and not Ep-CAM^neg^ breast cancer cell lines have cancer stem cell features including colony formation and tumorigenicity in NOD/SCID mice. Unfortunately, we could not characterize Ep-CAM^low^ primary cancer cells although they are interesting population as they correspond to the mammary stem cell enriched population in the normal mammary gland. This is because cancer cells, with Ep-CAM^low^/CD49f + phenotype, were present only in some patients and represented small percentages making them practically infeasible for us to study them.

The cell of origin of most of breast cancers still remains unknown. Keller et al [30] have demonstrated that transforming Ep-CAM^high^ luminal cells produce breast cancers commonly seen in the clinic, while transforming normal mammary basal Ep-CAM^low^/CD10+ cells produces a rare undifferentiated metaplastic type of breast cancer. Our results illustrate a similarity of phenotype between CSC and normal luminal progenitors. In addition, the phenotype of cancer cell lines being Ep-CAM^high^/CD49f + might further suggest the origin of CSC from Ep-CAM^high^/CD49f + normal luminal progenitors, at least in some cases of breast cancer. In agreement, Lim et al [11] and Molyneux et al [31] have demonstrated that the cell of origin of BRCA1 defective breast carcinomas, a predominantly triple negative type of breast cancer, is also in the Ep-CAM^high^/CD49f + luminal progenitor cells. Similarly, Lo et al [32] has shown the Ep-CAM^high^/CD49f + cells are the cell of origin of Her2/neu mouse model.

On the other hand, histological observation of the abundance of CD44^high^/CD24^low^ cells in the normal basal layer of the breast (anatomically in close contact with basement membrane) [33] has encouraged others to speculate that CD44^high^/CD24^low^ cancer cells might originate from the Ep-CAM^low^ normal basal layer [34]. Our results suggest that the luminal layer might be an additional source for CD44^high^/CD24^low^ cancer cells. This is further supported by our finding that only the luminal fraction of CD44^high^/CD24^low^ cells overlaps with ALDH^high^ cells. Overall, underscores the importance of using stem cell markers CD44^high^/CD24^low^ in combination with Ep-CAM/CD49f.

## Conclusions

**Figure 5:**
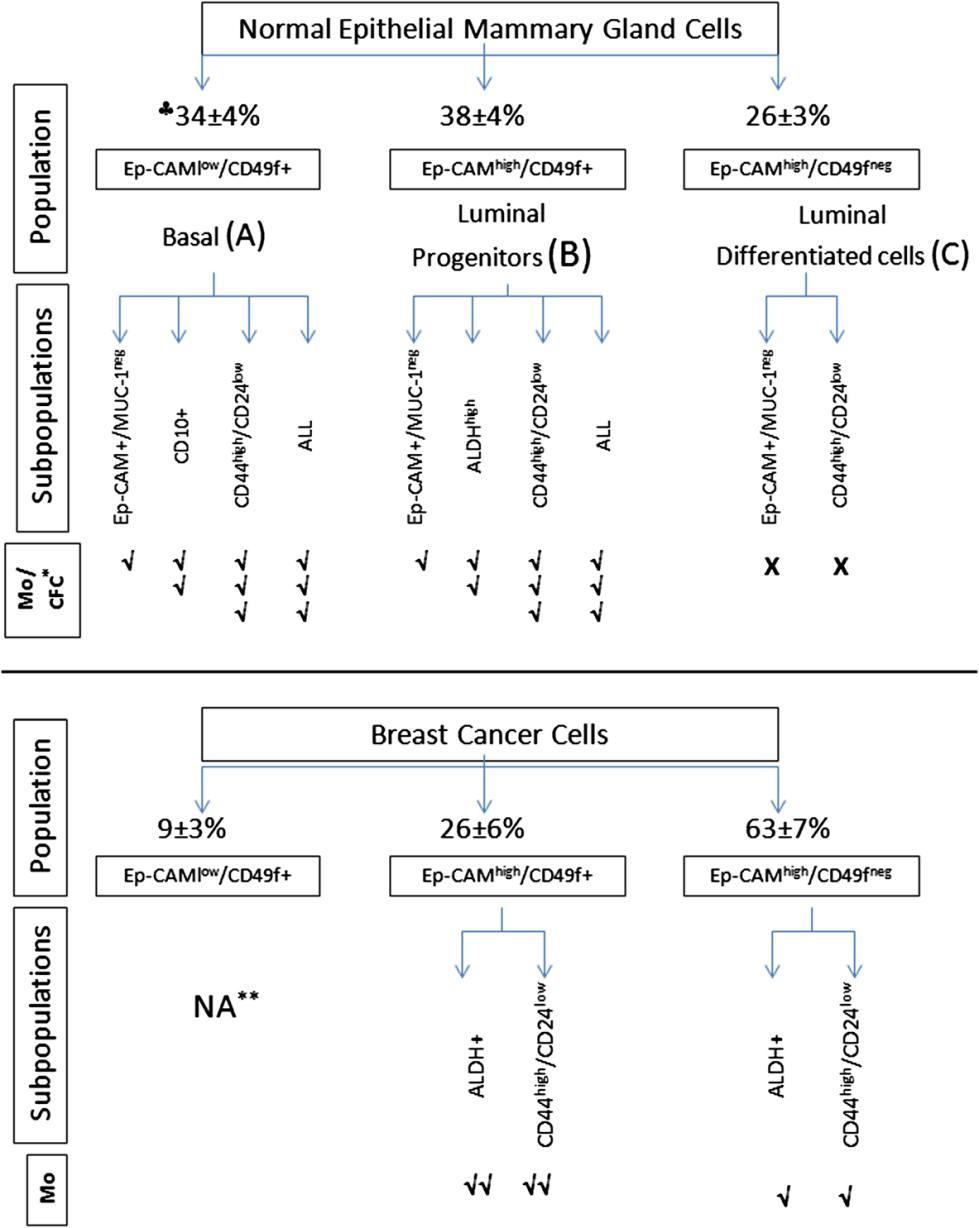
**Similarities/differences between normal and malignant breast epithelial stem/progenitor subpopulations.** The diagram summarizes the similarities and differences between the different Ep-CAM/CD49f populations. Each epithelial population was further fractionated into subpopulations based on the expression of other stem/progenitor cell makers. The three Ep-CAM/CD49f epithelial cell populations of the normal breast (**A**, **B**, and **C**), and their subpopulations, on top are compared with their malignant counterpart below. ^♣^Percentage of each epithelial population (average ± SEM, n = 9 normal & n = 12 for breast cancer).**NA = not done due to very low cell yield *Mo/CFC = mammosphere/colony forming cells. For mammosphere and colony forming ability, √√√ = high, √√ = medium, √ = low, X = none.

In the normal human mammary gland we have validated and compared side-by-side many breast stem/progenitor cell markers and found that among them only ALDH^high^, CD10+, CD44^high^/CD24^low^ Ep-CAM+/MUC-1^neg^ can enrich for stem/progenitor cells over CD49f + alone. CD44^high^/CD24^low^ had the highest ability to enrich for cell progenitors when used in combination with Ep-CAM/CD49f antibodies in order to differentiate between basal or luminal progenitors. Similarly, in breast cancer CD44^high^/CD24^low^ (as well as ALDH^high^) showed the highest ability to enrich for CSC. When normal breast stem/progenitor populations are compared with their counterparts in breast cancer, there were similarities and differences between stem/progenitor cells in normal and malignant breast. In both normal and cancer cells there was a correlation between CD44^high^/CD24^low^ phenotype and estrogen receptor negative status. In addition, there were cells with the CD44^high^/CD24^low^ phenotype in both Ep-CAM^high^/CD49f + and Ep-CAM^high^/CD49f^neg^ cell populations. Furthermore, ALDH was highly expressed by Ep-CAM^high^/CD49f + cells in both normal and malignant cells. On the other hand, breast cancer cells had mainly luminal phenotype, with an increase in the CD49f^neg^ fraction compared with normal breast which exhibited balanced populations of luminal (both differentiated and progenitor) and basal cells. The CD49f^neg^ cells in normal breasts could not form mammospheres, while in malignant breast they formed mammospheres, albeit to a much lesser extent than CD49f + cells (summarized in Figure 5). CD44^high^/CD24^low^ is expressed by both CD49f + and CD49f^neg^ cancer cells. However, CD44^high^/CD24^low^/CD49f + had significantly higher stem/progenitor ability as measured by mammosphere formation thus proposing that these cells are the best phenotype to identify breast CSC.

These findings may provide a better understanding of how CSC evolve, and which population to target and monitor during therapy, a leading step to eradicate this disease at its root.

## Abbreviations

ALDH: Aldehyde dehydrogenase; CFA: Colony forming assay; CSC: Cancer stem cell; ER: Estrogen receptor; PR: Progesterone receptor; FSC: Forward light scatter; SSC: Side light scatter; FSC-W: Forward light scatter width.

## Competing interests

All authors declare no conflict of interest.

## Authors’ contribution

HG: Conception and design, collection and assembly of data, data analysis and interpretation, manuscript writing, GS: collection and/or assembly of data, data analysis and interpretation, PM, AA, EB and FA: Collection and/or assembly of data, KA (plastic surgeon): Design and coordination/selection of patients' samples, CA: Conception and design, supervision of data analysis and interpretation and final approval/revision of manuscript. ALL authors read and approved the manuscript. All contributing authors approve the submission of this version of the manuscript and assert that the document represents valid work. All contributing authors have no disclosures to make.

## Pre-publication history

The pre-publication history for this paper can be accessed here:

http://www.biomedcentral.com/1471-2407/13/289/prepub

## Supplementary Material

Additional file 1: Figure S1 Gating strategy to analyze breast cells. Dot plot for isolated breast cells analyzed with sequential gating starting first with Forward scatter (FSC) and side scatter (SSC) to extract cells from debris followed by Relation of the area under the curve of the forward scatter signal (FSC-A) and the width of the forward scatter signal (FSC-W) to select for single cells only. DAPI positive cells were excluded to gate viable cells only. CD45 were used to exclude hematopoietic cells followed by gating on the different Ep-CAM/CD49 epithelial fraction. Finally the stem/progenitor subpopulation (Ep-CAM+/MUC-1^neg^, CD10+ or CD44^high^/CD24^low^) was sorted. With each stem/progenitor cells the remaining bulk from the specific epithelial Ep-CAM/CD49f fraction was also concomitantly sorted (identified as Rest). * Whenever necessary CD31+ endothelial cells were depleted by MACS prior cell acquisition. Click here for file

Additional file 2: Figure S2 Gating of different markers using antibody isotype control and or DEAB inhibitor of ALDH activity. A representative dot plot showing the background fluorescence of cells stained with either antibody isotype control or treated with DEAB inhibitor for ALDH activity. Click here for file

Additional file 3: Figure S3 Characterization of the Ep-CAM/CD49f four populations. A representative dot plot of normal mammary cells showing the three main epithelial Ep-CAM/CD49f populations designated as A, B and C in addition to the mesenchymal fraction. Sorted populations A and B contained cells that formed (*in vitro*) typical basal (myoepithelial) or luminal colonies respectively. Population C did not form colonies while mesenchymal (Ep-CAM^neg^/CD49f^neg^) cells formed typical mesenchymal-shaped colonies. Click here for file

Additional file 4: Figure S4 Expression of Stem/progenitor cell markers in Ep-CAM^high^/CD49f^neg^ cells. A representative dot plot showing the expression of each stem/progenitor cell marker in population C (Ep-CAM^high^/CD49f^neg^ cells) as analyzed by flow cytometry *numbers in brackets indicates average percentage (n = 5) ± SEM. Click here for file

Additional file 5: Figure S5 Mammosphere formation of cells positive for the above markers sorted from either population A (Ep-CAM^low^/CD49f+, top) or population B (Ep-CAM^high^/CD49f+, bottom) cell populations, error bars indicates mean ± SEM (n = 2). Mammospheres formed were normalized to unfractionated population A or B respectively. Click here for file

Additional file 6: Figure S6 Stem/progenitor subpopulations in breast cancer cell lines. A) Sketch that summarizes the Ep-CAM/CD49f profile of 9 commonly used breast cancer cell lines. B) Expression level of stem/progenitor cell markers in breast cancer cell lines, as determined by flow cytometry. Click here for file
