# Supplementary figures and images for: Profiling of normal and malignant breast tissue show CD44high/CD24low phenotype as a predominant stem/progenitor marker when used in combination with Ep-CAM/CD49f markers
Source: BMC Cancer. 2013 Jun 14;13:289. doi: 10.1186/1471-2407-13-289 (PMC3702414; doi:10.1186/1471-2407-13-289)

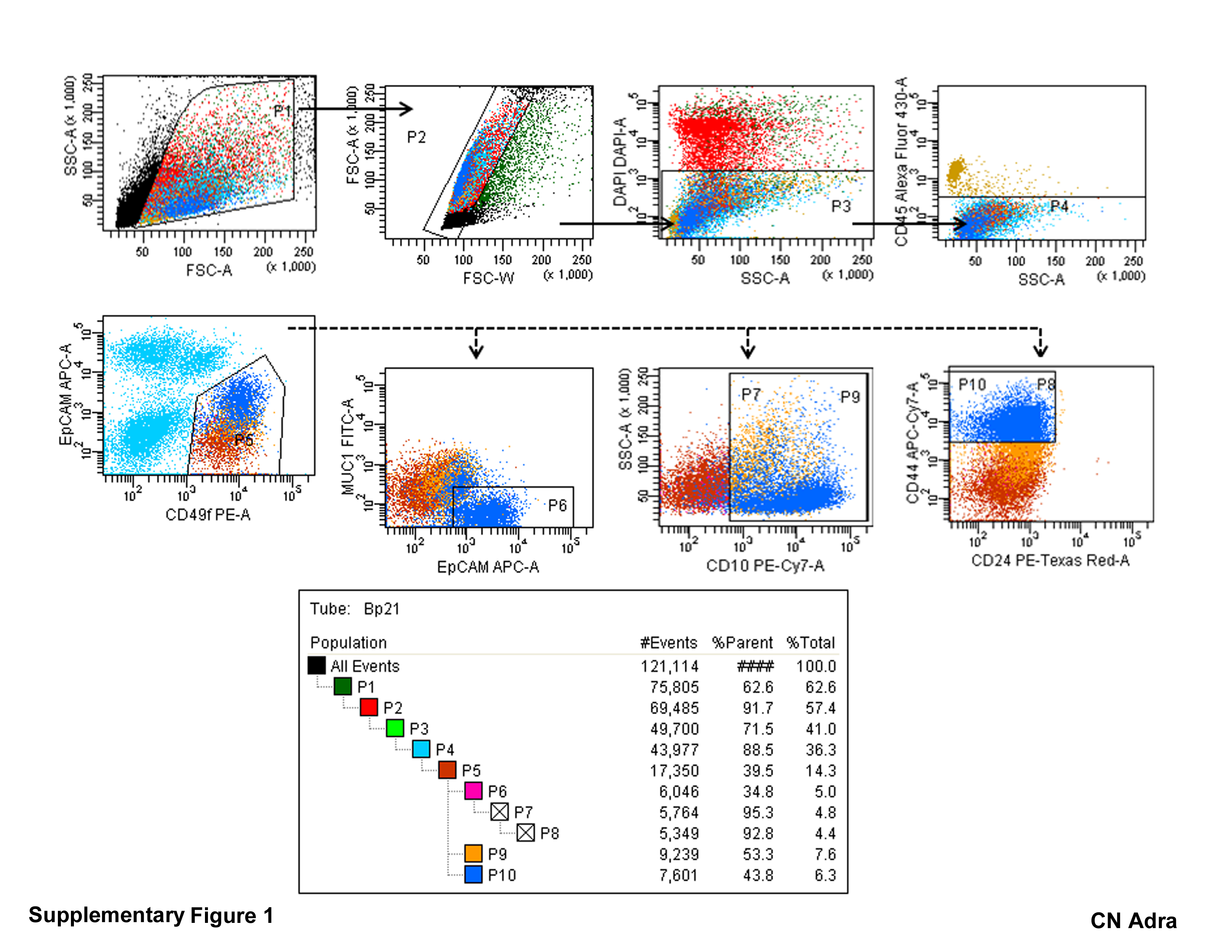

Supplement: Additional file 1: Figure S1 — Gating strategy to analyze breast cells. Dot plot for isolated breast cells analyzed with sequential gating starting first with Forward scatter (FSC) and side scatter (SSC) to extract cells from debris followed by Relation of the area under the curve of the forward scatter signal (FSC-A) and the width of the forward scatter signal (FSC-W) to select for single cells only. DAPI positive cells were excluded to gate viable cells only. CD45 were used to exclude hematopoietic cells followed by gating on the different Ep-CAM/CD49 epithelial fraction. Finally the stem/progenitor subpopulation (Ep-CAM+/MUC-1neg, CD10+ or CD44high/CD24low) was sorted. With each stem/progenitor cells the remaining bulk from the specific epithelial Ep-CAM/CD49f fraction was also concomitantly sorted (identified as Rest). * Whenever necessary CD31+ endothelial cells were depleted by MACS prior cell acquisition. [file 1471-2407-13-289-S1.tiff]

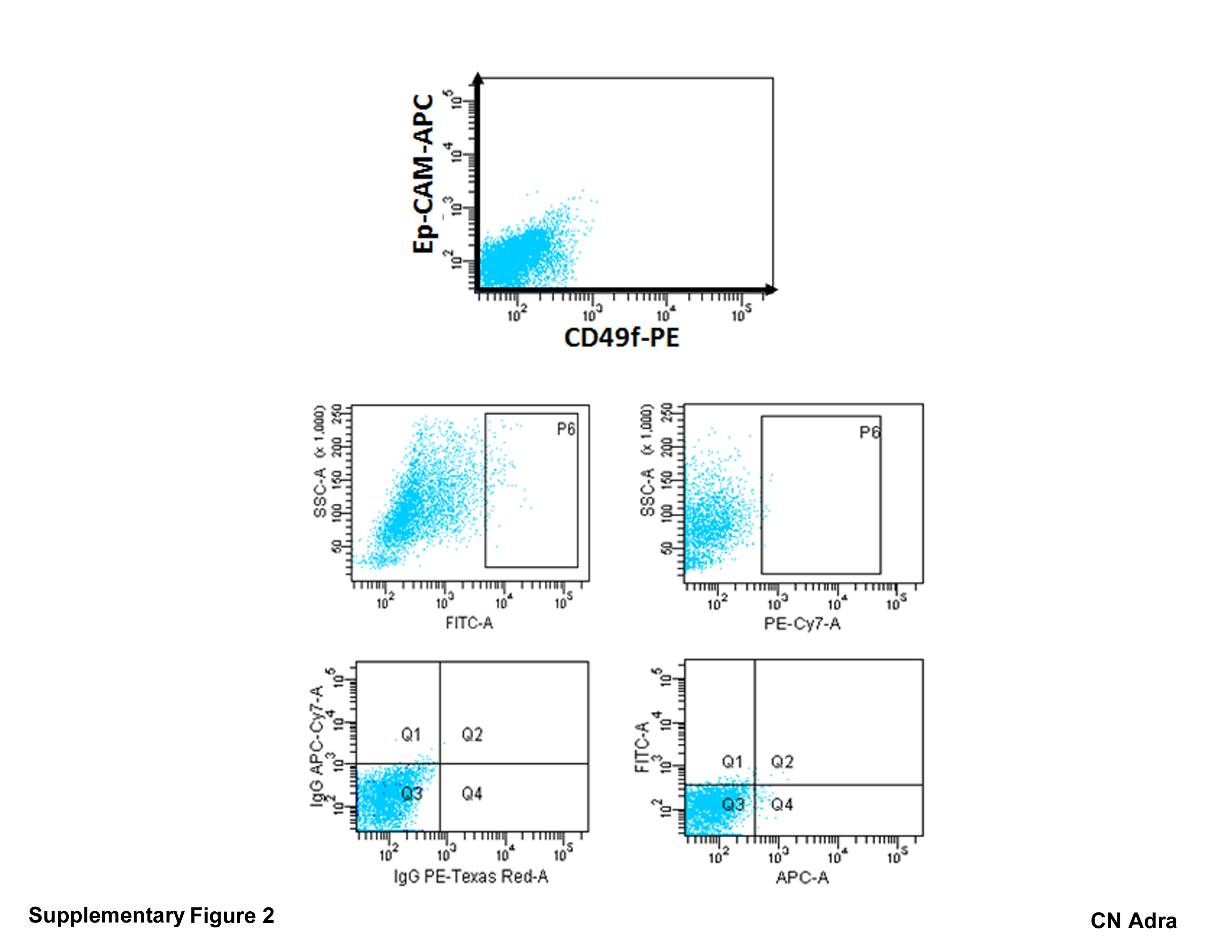

Supplement: Additional file 2: Figure S2 — Gating of different markers using antibody isotype control and or DEAB inhibitor of ALDH activity. A representative dot plot showing the background fluorescence of cells stained with either antibody isotype control or treated with DEAB inhibitor for ALDH activity. [file 1471-2407-13-289-S2.tiff]

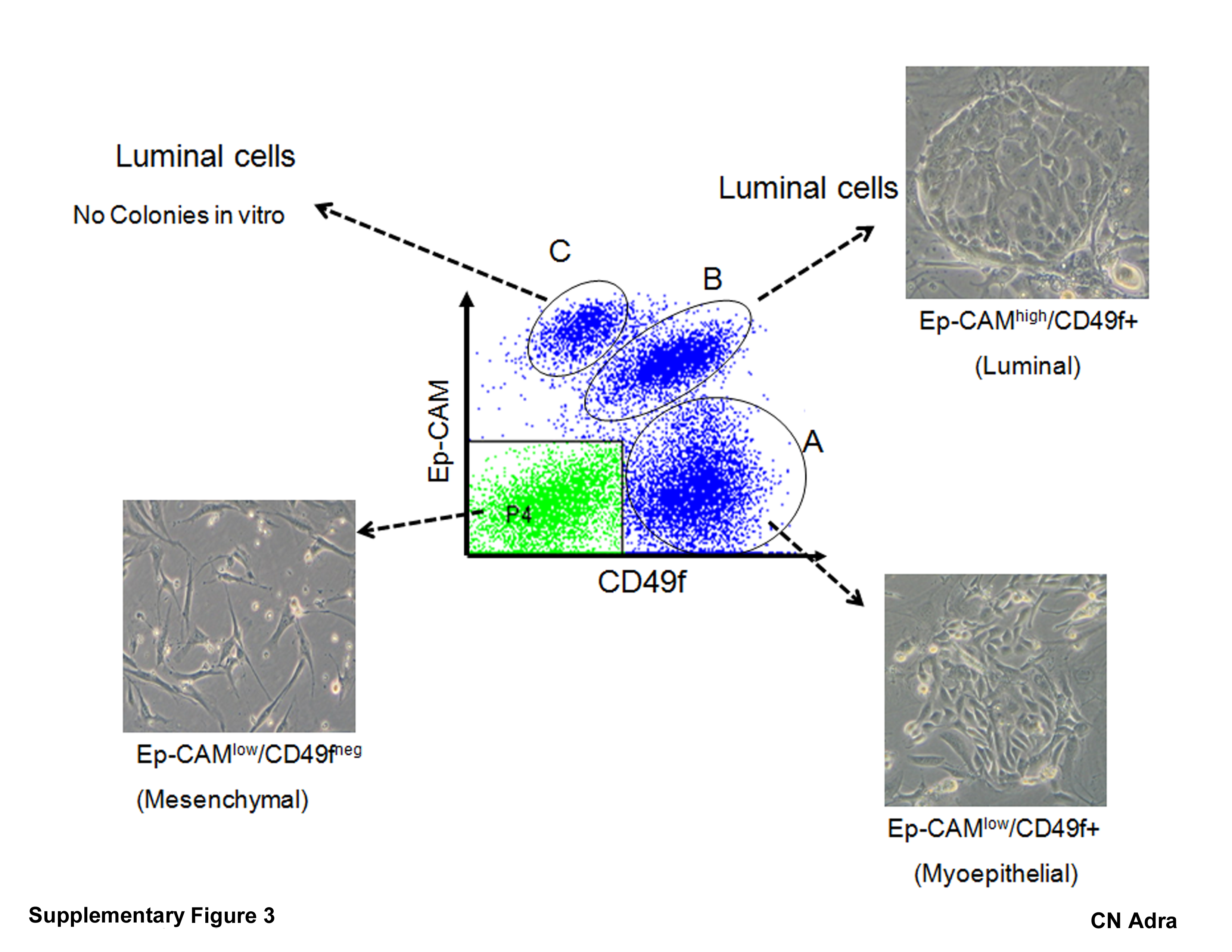

Supplement: Additional file 3: Figure S3 — Characterization of the Ep-CAM/CD49f four populations. A representative dot plot of normal mammary cells showing the three main epithelial Ep-CAM/CD49f populations designated as A, B and C in addition to the mesenchymal fraction. Sorted populations A and B contained cells that formed (in vitro) typical basal (myoepithelial) or luminal colonies respectively. Population C did not form colonies while mesenchymal (Ep-CAMneg/CD49fneg) cells formed typical mesenchymal-shaped colonies. [file 1471-2407-13-289-S3.tiff]

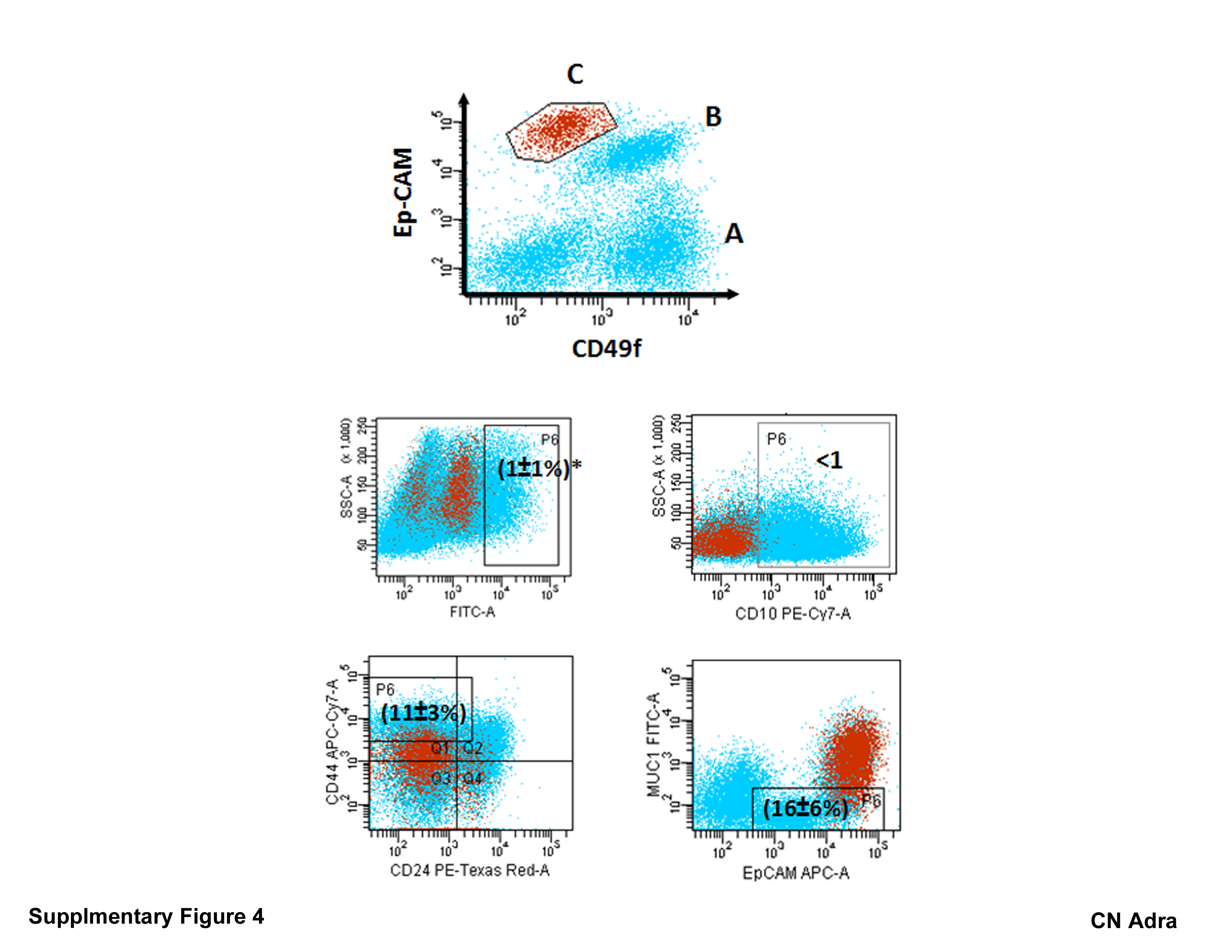

Supplement: Additional file 4: Figure S4 — Expression of Stem/progenitor cell markers in Ep-CAMhigh/CD49fneg cells. A representative dot plot showing the expression of each stem/progenitor cell marker in population C (Ep-CAMhigh/CD49fneg cells) as analyzed by flow cytometry *numbers in brackets indicates average percentage (n = 5) ± SEM. [file 1471-2407-13-289-S4.tiff]

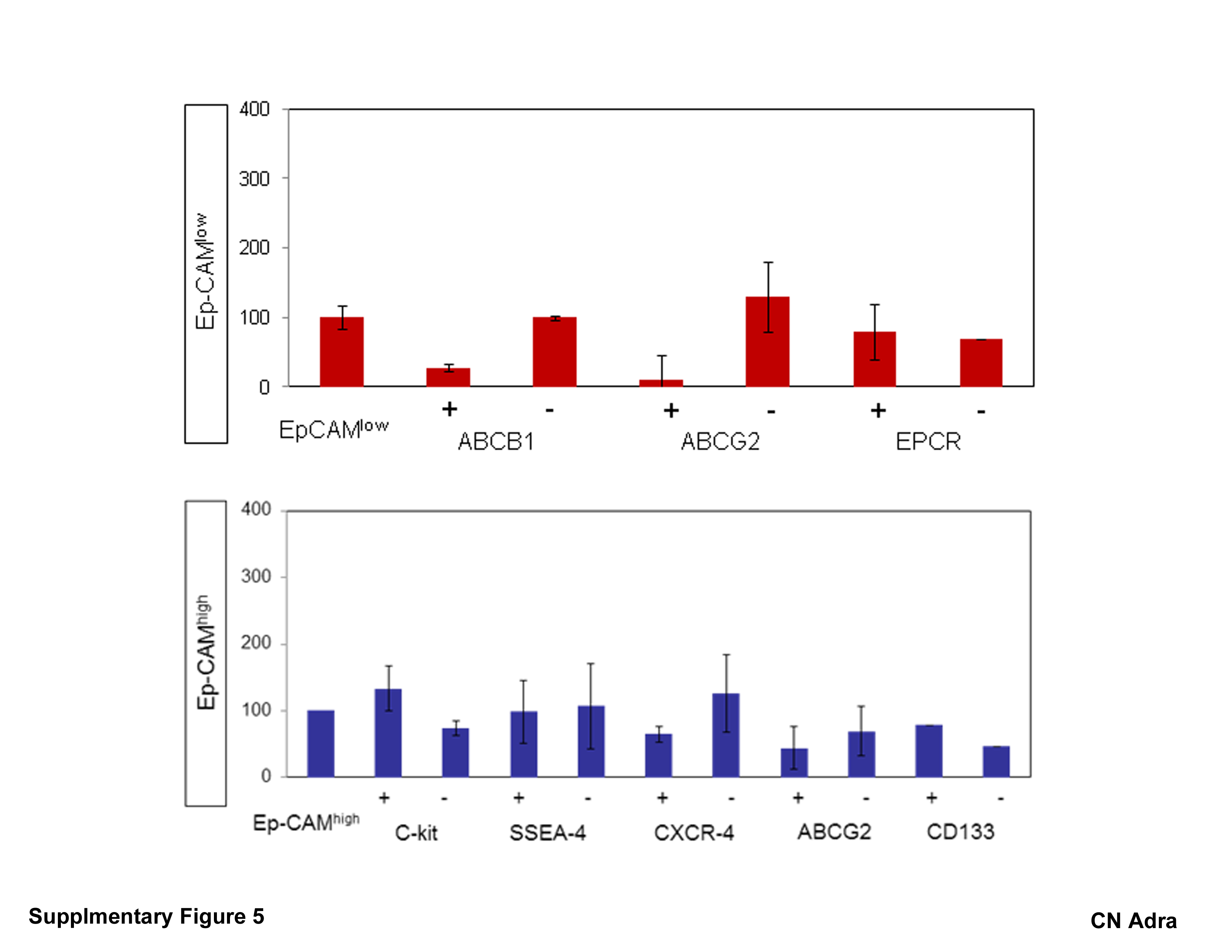

Supplement: Additional file 5: Figure S5 — Mammosphere formation of cells positive for the above markers sorted from either population A (Ep-CAMlow/CD49f+, top) or population B (Ep-CAMhigh/CD49f+, bottom) cell populations, error bars indicates mean ± SEM (n = 2). Mammospheres formed were normalized to unfractionated population A or B respectively. [file 1471-2407-13-289-S5.tiff]

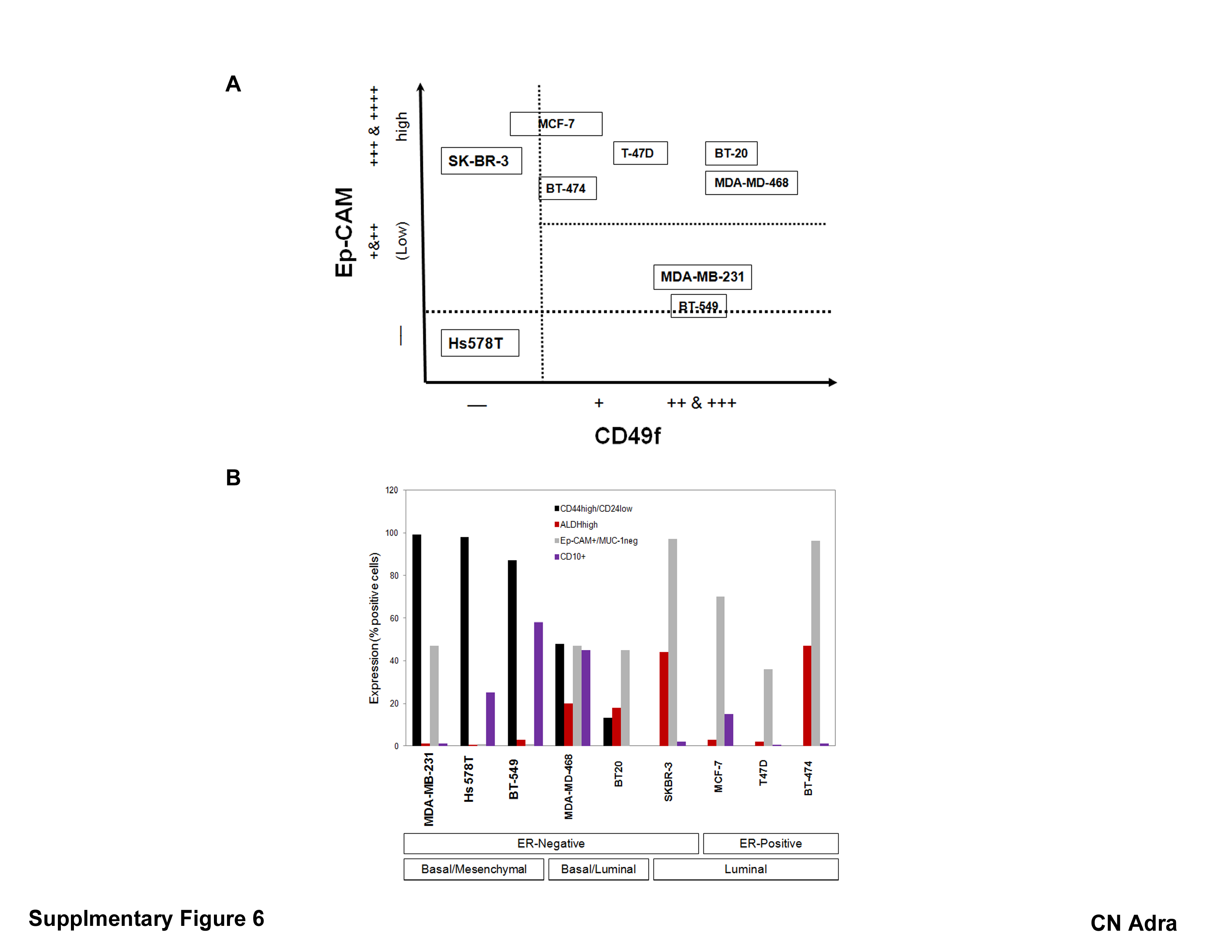

Supplement: Additional file 6: Figure S6 — Stem/progenitor subpopulations in breast cancer cell lines. A) Sketch that summarizes the Ep-CAM/CD49f profile of 9 commonly used breast cancer cell lines. B) Expression level of stem/progenitor cell markers in breast cancer cell lines, as determined by flow cytometry. [file 1471-2407-13-289-S6.tiff]
